# SF1, a Standardised Fraction from *Clinacanthus nutans* Modulates Notch1 Signalling in Cervical Cancer Stem-Like Cells

**DOI:** 10.21315/mjms-04-2025-279

**Published:** 2025-10-31

**Authors:** Faridah Ismail, Yusmazura Zakaria, Muhammad Lokman Md Isa, Nik Fakhuruddin Nik Hassan, Suat Cheng Tan

**Affiliations:** 1 Department of Basic Medical Sciences, Kulliyyah of Medicine, International Islamic University Malaysia, Kuantan, Pahang, Malaysia; 2 Biomedicine Programme, School of Health Sciences, Health Campus, Universiti Sains Malaysia, Kubang Kerian, Kelantan, Malaysia; 3 Institute of Planetary Survival for Sustainable Well-being (PLANETIIUM), International Islamic University Malaysia, Kuantan, Pahang, Malaysia; 4 Forensic Science Programme, School of Health Sciences, Health Campus, Universiti Sains Malaysia, Kubang Kerian, Kelantan, Malaysia

**Keywords:** Clinacanthus nutans, cancer stem cells, cervical cancer, Notch1 signalling, stemness

## Abstract

**Background:**

Aberrant activation of Notch1 signalling in Cervical Cancer Stem Cells (CCSCs) plays a key role in the disease development, progression, metastasis, recurrence, and chemoresistance. Thus, targeting Notch1 is crucial for CCSC eradication. SF1, a standardised fraction from *Clinacanthus nutans (C. nutans)*, has shown potent cytotoxic effects against cervical cancer cells, including CCSCs. However, its mechanism is uncertain. This study aimed to elucidate whether SF1 can inhibit Notch1 signalling in CCSCs.

**Methods:**

SF1 was isolated from *C. nutans* leaves using a bioassay-guided fractionation. For CCSC enrichment, the human cervical cancer cell line SiHa was grown as non-adherent cells in stem cell-conditioned media (cervospheres) and characterised using sphere formation assay and flow cytometric analysis of stemness markers CD49f, CK17, Sox2, Nanog, and Oct4. The cells were then subjected to SF1 treatment, and Notch1 activity was examined using Western blot and quantitative RT-PCR.

**Results:**

The study discovered that SiHa cervospheres were efficient at forming spheres and contained more Sox2- and Nanog-positive cells than SiHa monolayers. In addition, cervospheres exhibited elevated Notch1 activity, with higher levels of the active *Notch1* intracellular domain (NICD) protein and Notch1 mRNA than their monolayer counterparts. Following SF1 treatment, NICD protein levels in the cervospheres were significantly reduced, while Notch1 mRNA levels increased. These findings indicate that SF1 modulates *Notch1* signalling at the post-transcriptional or post-translational level. However, the precise mechanism remains to be elucidated.

**Conclusion:**

SF1 possesses antitumor effects against SiHa-derived CCSCs via modulation of Notch1-signalling, a pathway associated with Cancer Stem Cells stemness.

## Introduction

Cervical cancer has been recognised as one of the most common malignancies of the female reproductive organs globally (1). Despite the advances in cervical cancer prevention and diagnosis, recent reports have highlighted a rise in the incidence of advanced-stage cervical cancer (2). The challenges that arise in the management of cervical cancer, including poor treatment outcomes, cancer recurrence, metastasis, and multiple drug resistance, can be elucidated by the presence of Cancer Stem Cells (CSCs) (3). CSCs refer to a minor subset of cancer cells within a tumour that possess stem and progenitor cell properties, including unlimited self-renewal and differentiation capacity to give rise to the heterogeneous phenotype of the tumour cells. Apart from that, CSCs possess unique properties and an extensive array of survival mechanisms. They can evade conventional cancer therapy by exhibiting a high tumorigenicity and metastatic potential, multidrug resistance, epigenetic reprogramming, and tumour microenvironment protection (4, 5). Hence, developing novel non-toxic drug therapies that effectively target both CSCs and proliferating cancer cells is crucial.

Emerging evidence indicates that Notch signalling is crucial for maintaining the survival of CSCs. Notch signalling is a type of direct intercellular communication that plays a crucial role in regulating normal stem cell proliferation, cell fate determination, and apoptosis during embryonic development and throughout the entire lifespan. However, it is discovered to be deregulated in CSCs, leading to uncontrolled self-renewal, differentiation, and tumorigenesis. In mammals, there are four Notch receptors (Notch1 through 4) and five classic ligands termed Jagged (JAG)1 and JAG2, and Delta-like (DLL)1, DLL3, and DLL4 (6, 7). Among the Notch receptor family members, Notch1 is primarily implicated in cancers. It has been extensively reported for its pro-oncogenic effects (8). In cervical cancer, aberrant Notch1 signalling has been closely linked to disease initiation and progression (9). Furthermore, the upregulation of Notch1 was evident in the Cervical CSC (CCSC) population, which was associated with preserving their stemness properties (10). Overexpression of Notch1 was also correlated with a poor prognosis and increased metastases in cervical cancer patients (11). Hence, Notch1 is considered a promising therapeutic target for CCSCs.

*Clinacanthus nutans* (Burm. f.) Lindau (*C. nutans*), a Acanthaceae tropical herb, is a popular Southeast Asian and Chinese herbal remedy. It has been used for ages to treat inflammation, diabetes, and cancer (12). Several studies have demonstrated that *C. nutans* potently inhibits various cancer cell types (13–15). SF1, a standardised, semi-purified fraction derived from *C. nutans* leaf extract, has been identified as contributing to its anticancer properties. SF1 has been documented to demonstrate selective cytotoxicity against cervical cancer cells by inducing apoptosis via the suppression of the G1/S cell cycle checkpoint and to exert a suppressive effect on cervical tumour growth *in vivo* (15–17). Furthermore, our prior research revealed that SF1 may target CCSCs by inhibiting their viability, self-renewal, sphere formation, stemness marker expression, and tumour growth in mice (18). However, the mechanism underlying this inhibition is unclear. Therefore, this work further examined the anticancer mechanisms of SF1 on CCSC-like cells, focusing on its inhibition of Notch1 signalling.

## Methods

### Cell Culture

Human cervical cancer cell line SiHa (ATCC@-HTB-35, squamous cell carcinoma, HPV-16) was acquired from the American Type Culture Collection (ATCC, USA). The monolayer cells were maintained in Dulbecco’s Modified Eagle Medium (DMEM, Nacalai Tesque, Japan) supplemented with 10% Fetal Bovine Serum (FBS, Sigma-Aldrich, USA) and 100 U/ml penicillin/streptomycin (Nacalai Tesque, Japan). To enrich CCSC-like cells (cervospheres), the SiHa monolayer cells were cultured in a serum-free CSC conditioned medium using the nonadhesive culture system described by Chen et al. (19) with minor modifications. Briefly, the monolayer cells were grown to 70% to 80% confluence, harvested, washed with Phosphate-Buffered Saline (PBS) and cultured in Dulbecco’s Modified Eagle Medium/Nutrient Mixture F-12 (DMEM/F-12, Nacalai Tesque, Japan) supplemented with 20 ng/ml epidermal growth factor (Sigma-Aldrich, USA), 20 ng/ml basic fibroblast growth factor (Roche, USA), 10 μl/ml B27 (50X, Gibco®), and 100 U/ml penicillin/streptomycin (Nacalai Tesque, Japan) in 1.5% agarose coated 6-well plates at a density of 1 × 10^4^ cells/ml. The cells were cultured for seven to eight days at 37°C in a humidified incubator with 5% CO_2_. Cervosphere formation was monitored daily, and one-third of the media was replaced every three days.

### Sphere Formation Assay

The cervospheres were collected by gentle centrifugation at 300 × *g* for 5 min, washed, and dissociated to single cells using 0.05% trypsin. The cells were counted and replated in agarose coated 6-well plates at 1 × 10^4^ cells/ml in the CSC conditioned medium. The cells were incubated for seven days, and one-third of the media was replaced every three days. Over time, the number of each well with a diameter greater than 100 μm was manually counted using the inverted microscope. Sphere-Forming Efficiency (SFE) was calculated as the number of spheres divided by the number of seeded cells and expressed as a percentage. The cervospheres were serially propagated and expanded every seven days. The SFE was calculated from the first through the fifth generation.

### Analysis of Stemness Markers by Flow Cytometry

Cells from SiHa monolayers and cervospheres were collected separately, centrifuged, dissociated into single cells, and counted as previously mentioned. For each conjugated antibody staining, 1 × 10^6^ cells were incubated with anti-CD49f-PE (Miltenyi Biotec, Germany) and anti-CK17-FITC (Santa Cruz Biotechnology, USA) in flow buffer consisting of PBS (1X) with 0.5% Bovine Serum Albumin (BSA) for 30 min on ice in the dark. For incubation with fluorescence-conjugated antibodies that recognise Sox2, Nanog, or Oct4, cells were fixed with 4% paraformaldehyde (Nacalai Tesque, Japan) for 10 min at room temperature, followed by permeabilisation with 0.1% Triton X-100 (Sigma-Aldrich, USA) in PBS for 15 min at room temperature. Permeabilised cells were washed with flow buffer (PBS containing 1% BSA) and incubated with anti-SOX2-AlexaFluor488 (BioLegend, USA), anti-OCT4-PE, and anti-NANOG-PE (both Miltenyi Biotec, Germany) for 30 min on ice in the dark. After incubation, cells were washed with flow buffer, pelleted by centrifugation at 300 × g for 5 min, and resuspended in PBS for acquisition on a BD FACScan™ flow cytometer. At least 10,000 events were recorded per sample, and data were analysed using FlowJo™ v10 software.

### Plant Materials and Extraction of SF1

The aerial parts of *C. nutans* were collected from Pengkalan Chepa, Kelantan, and a voucher specimen number PIIUM 0238-2 was deposited at the Herbarium of the Kulliyyah of Pharmacy, International Islamic University Malaysia. The extraction was performed in accordance with the published protocol (15). The leaves were dried, ground to a coarse powder, and sequentially extracted with hexane and chloroform (HMBG, Germany). The extract was concentrated in vacuo at 40°C to give the dried chloroform extract. Dry column vacuum chromatography was used to chromatograph the chloroform extract on silica gel 60 (250 g) (Merck, Germany). Hexane-ethyl acetate (1:1) (Merck, Germany) was used to elute the column and isolate the active fraction, namely F11. The SF1 fraction was isolated by loading the F11 fraction onto the second chromatography column with acetonitrile-methanol (2:8) (Merck, Germany) as the elution solvent. The collection of both F11 and SF1 fractions was monitored by Thin-Layer Chromatography (Merck, Germany) using chloroform-methanol (1:1) and chloroform-methanol (2:8) as the mobile phases, respectively. Retention factor (Rf) was calculated using the formula: Rf = distance moved by solute/distance moved by solvent.

### Cell Treatments

SF1 was dissolved in Dimethyl sulfoxide (DMSO, Nacalai Tesque, Japan) and diluted in fresh CSC culture media before cell treatment. Cervospheres cultured for seven days were treated with 17.07 μg/ml SF1 for 72 hours. Cisplatin at 4.29 μg/ml (Sigma-Aldrich, USA) was used as the positive control and DMSO at less than 0.1% as the negative control, both used for treatment over 72 hours. The concentrations of cisplatin and SF1 were determined by the cytotoxicity assays conducted in the preceding study (18).

### Western Blot Analysis

Total proteins from SiHa monolayers and cervospheres treated or untreated were extracted using Radioimmunoprecipitation Assay (RIPA) buffer supplemented with a complete Ethylenediaminetetraacetic Acid (EDTA)-free protease inhibitor cocktail (both Solarbio, China) and incubated on ice for 30 min. The protein concentration was determined by the modified Bradford quantification method (Solarbio, China). Note that 50 μg of each protein sample was boiled in Sodium Dodecyl Sulfate (SDS)-containing sample buffer (Bio-Rad, USA), separated by 7.5% SDS polyacrylamide gel electrophoresis (SDS-PAGE), and transferred onto Polyvinylidene Difluoride (PVDF) membranes by semi-dry blotting. Membranes were blocked with 5% non-fat milk in PBS containing 0.1% Tween-20 for 1 hour. Consequently, the membranes were incubated overnight at 4°C with Notch1 antibody (1:300, clone A-8; Santa Cruz Biotechnology, USA) that recognises a C-terminal epitope present in the cleaved Notch1 intracellular domain (NICD; ~114 kDa). This antibody does not detect the full-length ~300 kDa Notch1 receptor, serving as a marker of Notch1 activation. β-actin antibody (1:1000, Santa Cruz Biotechnology, USA) was the loading control. Antibody recognition was detected with Horseradish Peroxidase (HRP)- conjugated secondary antibodies, M-IgGƙ BP-HRP (1:2000) and M-IgG Fc BP-HRP (1:10000) (both Santa Cruz Biotechnology, USA), for Notch1 and β-actin antibodies, respectively. The proteins were visualised using Chemi-Lumi One L (Nacalai Tesque, Japan) and Gel Doc imaging systems for enhanced chemiluminescence. Densitometry was performed using ImageJ software, and chemiluminescence was normalised to β-actin protein levels.

### Real Time Quantitative Polymerase Chain Reaction Analysis (RT-qPCR)

RNA extraction of SiHa monolayers and cervospheres was performed using the innuPREP RNA Mini Kit 2.0 (Analytik Jena) following the manufacturer’s instructions. The RNA concentration and purity were measured using a NanoDrop ND-1000 spectrophotometer. 1 μg of RNA template from each sample was subjected to first-strand cDNA synthesis using the SensiFAST™ cDNA Synthesis Kit (Bioline, USA). By means of real time qPCR, the cDNA was evaluated to determine the expression of the *Notch1* gene (F: 5′-GCA GAG GCG TGG CAG ACT AT-3′ and R: 5′-CGG CAC TTG TAC TCC GTC A-3′) and the *GAPDH* gene (F: 5′-TCC AAA ATC AAG TGG GGC GA-3′ and R: 5′-ATG ACG AAC ATG GGG GCA TC-3′) as a control. The qPCR assay was run in a total volume of 20 μL per reaction, comprising 10 μL of SensiFAST™ SYBR No-ROX SYBR green (Bioline, USA), 0.8 μL of forward primer (400 nM), 0.8 μ L of reverse primer (400 nM), 6.4 μL of nuclease-free water, and 2 μ L of cDNA template (100 *n*g). No template controls were included to exclude the presence of DNA contamination. The expression of *Notch1* mRNA was quantified by real time PCR and normalised to the reference gene *GAPDH*. Fold change was calculated using the 2^−ΔΔ^Ct method, representing the normalised expression ratio in the treated or experimental group relative to the control group.

### Statistical Analysis

Statistical analysis was performed using GraphPad Prism 9.0 (GraphPad, USA). The Shapiro-Wilk normality test was employed to determine data distribution normality. An independent *t*-test or Mann-Whitney U test was used to determine significance for two data sets. For data with more than two groups, one-way Analysis of Variance (ANOVA) with post-hoc Tukey’s tests or Kruskal-Wallis with post-hoc Dunn’s tests were used to assess group differences. Data are presented as mean ± standard deviation (SD), with statistical significance set at *P* < 0.05 (*P < 0.05, **P < 0.01, ***P < 0.001, ***P < 0.0001).

## Results

### Sphere-forming Capacity of Cervospheres

**Figure 1:**
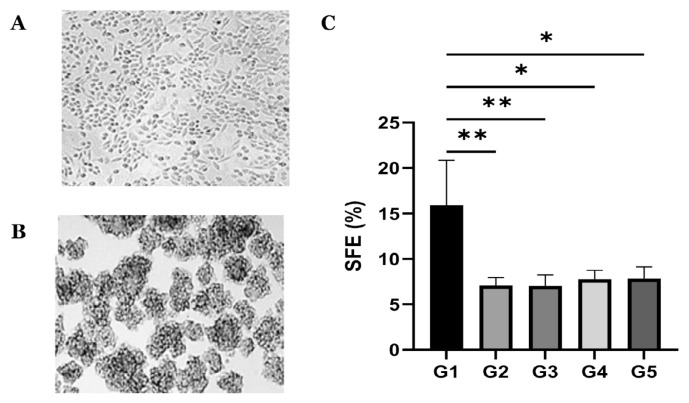
SiHa-derived cervospheres and their sphere formation efficiency: (A) SiHa cells in monolayer culture (×20 magnification); (B) SiHa cells grown as cervospheres (×20 magnification); (C) SFE of the cervosphere cells from G1 to G5. Data are expressed as mean ± SD (*n* = 3 independent assays) and analysed using one-way ANOVA with post-hoc Tukey Test; *P < 0.05; **P < 0.01 versus control (G1); G = Generation

Sphere formation assay was performed to evaluate the ability of SiHa-derived cervospheres to self-renew and form spheres. Figures 1A–1B show that the SiHa cell line, grown as non-adherent cells in CSC conditioned medium, led to the generation of anchorage-independent multicellular spheres (cervospheres) representing putative CCSCs. The cervospheres developed from the proliferation of single cells and progressively enlarged to form large and tightly compact spheres by day 7 of culture. Figure 1C indicates that the cervospheres were successfully propagated and maintained for at least five generations. The SFE was high in generation 1 (15.92 ± 4.93%, *P* < 0.05) but decreased by almost 50% in generations 2–5 cervospheres (7.09 ± 0.85%, 7.04 ± 1.20%, 7.78 ± 0.95%, and 7.85 ± 1.29%, respectively). Although the high SFE did not persist throughout the passages, the SFE rate remained relatively constant from the second through the fifth generation, with no sign of growth inhibition. These results suggest the ability of cervospheres to self-renew and sustain their undifferentiated state for the long term.

### Expression of Stemness Markers in Cervospheres

**Figure 2:**
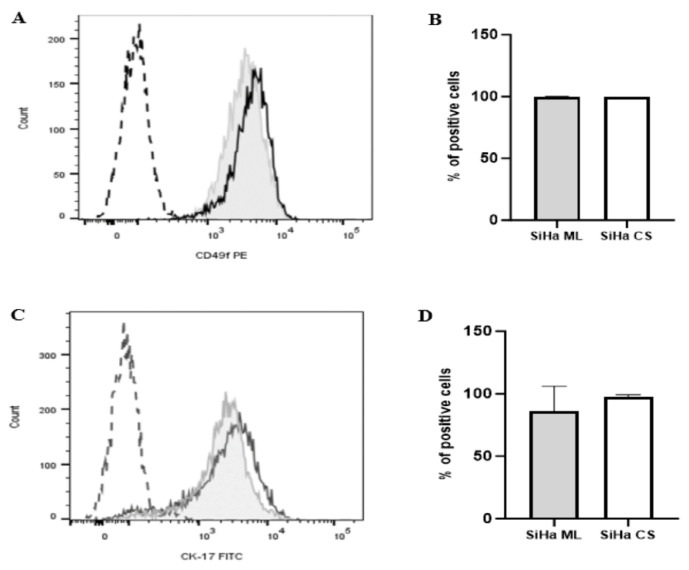
Flow cytometric analysis of CCSC phenotypic marker for characterisation of cells within cervospheres: (A, C) Histogram representation showing the percentages of CD49f- and CK17-positive cells in SiHa ML cells (tinted with line) and cervosphere cells (black line), respectively; Long dashes represent autofluorescence (unstained control); (B, D) Statistical analysis of the percentages of CD49f- and CK17-positive cells, respectively; Data are expressed as mean ± SD (*n* = 3 independent assays) and analysed using Mann-Whitney test (B) and independent *t*-test (D); SiHa ML = SiHa monolayers; SiHa CS = SiHa cervospheres

CD49f and CK17 represent CCSC surface biomarkers. Figures 2A–2B show that both SiHa monolayers and cervospheres displayed a high and comparable percentage of CD49f-positive cells (99.90 ± 0.10% and 99.93 ± 0.06%, respectively). In Figures 2C–2D, the percentage of CK17-positive cells was slightly higher in cervospheres (97.13 ± 2.06%) compared to monolayers (86.03 ± 19.88%). However, under the applied conditions, no significant difference in CD49f- and CK17-positive cell percentages was observed between the two culture types.

**Figure 3:**
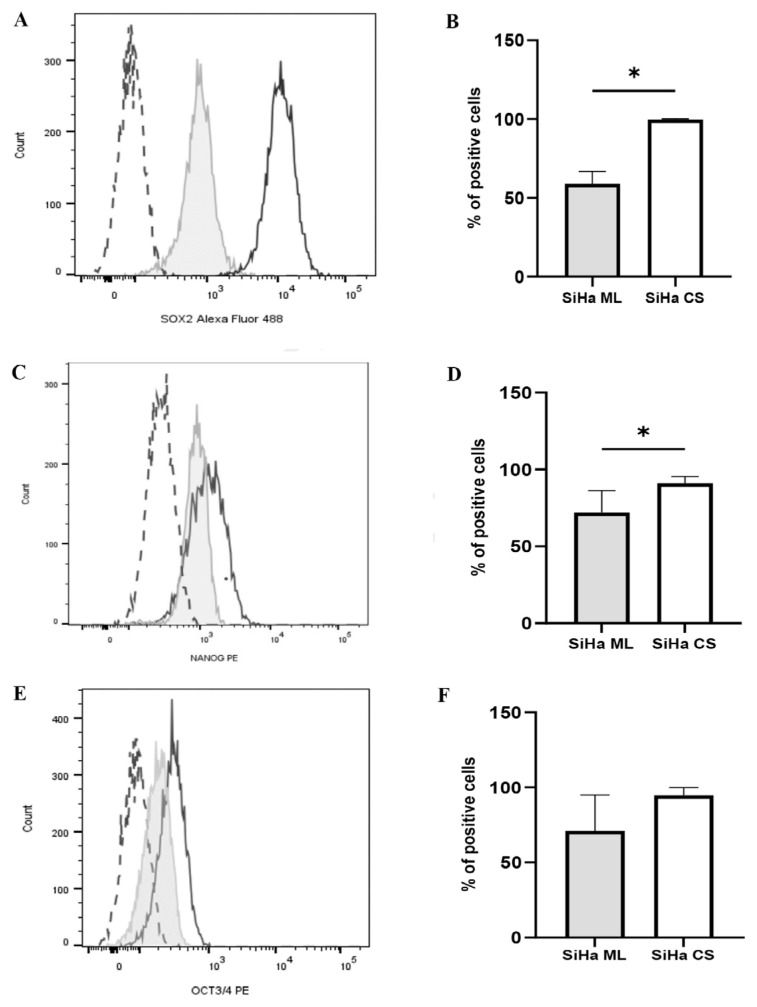
Flow cytometric analysis of CCSC pluripotent markers for characterisation of cells within cervospheres: (A, C, E) Histogram representation showing the percentages of Sox2-, Nanog-, and Oct4-positive cells in SiHa ML cells (tinted with a line) and cervosphere cells (black line), respectively; Long dashes represent autofluorescence (unstained control); (B, D, F) Statistical analysis of the percentages of Sox2-, Nanog-, and Oct4-positive cells, respectively; Data are expressed as mean ± SD (*n* = 3 independent assays) and analysed using Mann-Whitney test (B) and independent *t*-test (D, F); *P < 0.05 versus control (SiHa ML); SiHa ML = SiHa monolayers; SiHa CS = SiHa cervospheres

Sox2, Nanog, and Oct4 are transcription factors that indicate cell stemness. Figures 3A–3D show that the percentages of Sox2- and Nanog-positive cells increased significantly (*P* < 0.05) in cervospheres (99.70 ± 0.55% and 90.98 ± 4.42%, respectively) compared to monolayers (58.88 ± 7.99% and 71.93 ± 14.42%, respectively). The percentage of Oct4-positive cells in cervospheres (94.63 ± 5.15%) was also higher than in monolayers (70.90 ± 24.06%), although the difference was not statistically significant (Figures 3E–3F). The high percentages of pluripotent stemness marker-positive cells in the cervospheres indicate an enrichment of CCSC-like cells under stem cell– selective conditions.

### Effect of SF1 on Notch1 Protein Expression in Cervospheres

**Figure 4:**
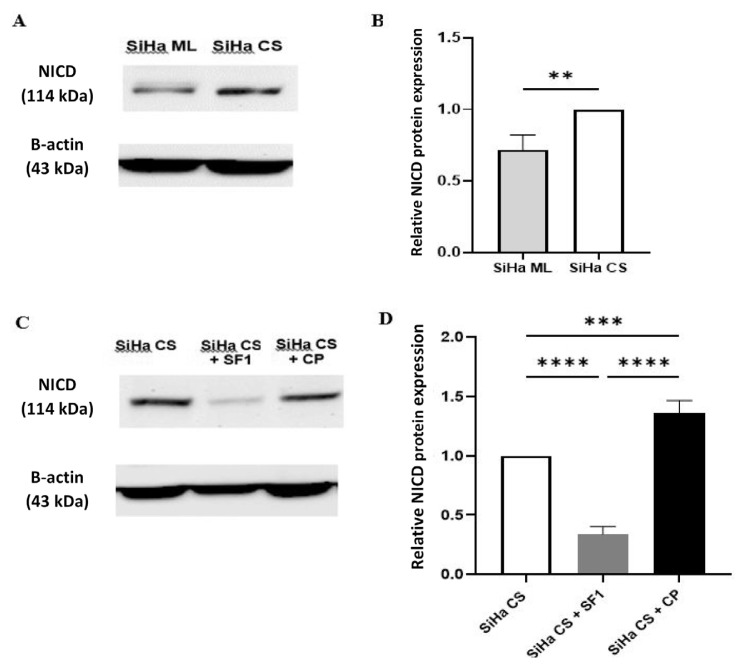
Western blot analysis of cleaved NICD expression: (A, B) Representative immunoblots and statistical analysis of the relative expression of NICD (~114 kDa) and β-Actin (~43 kDa, loading control) in untreated SiHa ML and SiHa CS; (C, D) Representative immunoblots and statistical analysis of NICD expression in SiHa CS treated with SF1 or CP; Data are expressed as mean ± SD (*n* = 3 independent assays) and analysed using independent *t*-test (B) and one-way ANOVA with post-hoc Tukey Test (D); **P < 0.01, ***P < 0.001, ****P < 0.0001 versus control (SiHa CS). SiHa ML = SiHa monolayers; SiHa CS = SiHa Cervospheres; CP = Cisplatin

Cleaved NICD represents the transcriptionally active form of Notch1 that translocates to the nucleus to regulate downstream target genes, directly indicating Notch1 signalling activation. As shown in Figures 4A–4B, the expression level of NICD in SiHa monolayers was decreased by 0.7-fold compared to the derived cervospheres. This result is expected given the previous discovery that NICD is upregulated in CCSCs, thus suggesting that our cervosphere culture is enriched in CCSCs. From Figures 4C–4D, it was observed that SF1 treatment of the cervospheres led to a substantial decrease in NICD expression by 0.3-fold compared to the untreated cervospheres (*P* < 0.0001). Additionally, the level of Notch1 expression in SF1-treated cervospheres was significantly lower than in cisplatin-treated cervospheres. Cisplatin treatment has resulted in a marked elevation of NICD expression within the cervospheres by 1.4-fold, relative to the untreated cervospheres (*P* < 0.001). Hence, the results suggest that the inhibitory effect of SF1 on the stemness properties of cervospheres, as demonstrated in prior research, may be mediated through Notch1 signalling.

### Effect of SF1 on Notch1 Gene Expression in Cervospheres

**Figure 5:**
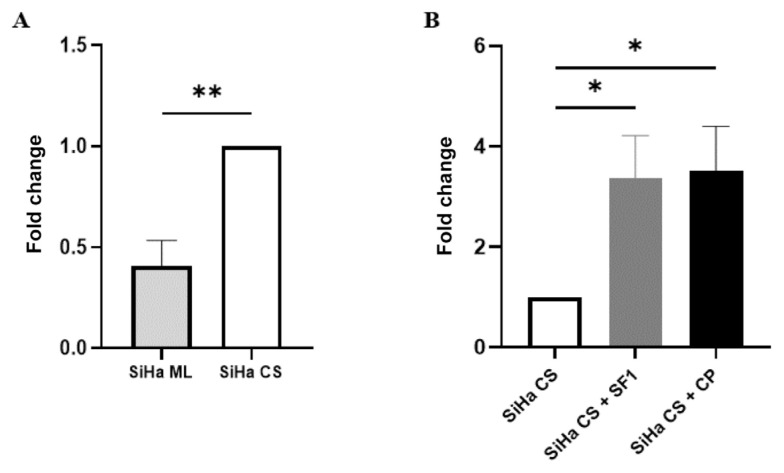
Real time qPCR analysis of *Notch1* mRNA expression: (A) *Notch1* mRNA expression in untreated SiHa ML and SiHa CS; (B) The effects of SF and CP on *Notch1* mRNA expression in cervospheres; Results are expressed as fold change relative to the control group (SiHa CS); Data are expressed as mean ± SD (*n* = 3 independent assays) and analysed using independent *t*-test (A) and one-way ANOVA with post-hoc Tukey Test (B); **P < 0.01, *P < 0.05 versus control (SiHa CS); SiHa ML = SiHa monolayers; SiHa CS = SiHa cervospheres; CP = cisplatin

The findings in Figure 5A indicate a statistically significant difference (*P* < 0.01) in the expression of *Notch1* mRNA between SiHa monolayer and cervosphere cultures. The monolayers exhibited a 0.4-fold decrease in *Notch1* mRNA expression relative to the cervospheres. On the other hand, *Notch1* mRNA expression was significantly upregulated following SF1 treatment of the cervospheres, showing a 3.4-fold increase in comparison to the untreated cervospheres (*P* < 0.05) (Figure 5B). Similarly, cisplatin treatment was found to elevate the level of *Notch1* mRNA expression in the cervospheres by 3.5-fold, relative to the untreated cervospheres (*P* < 0.05). Hence, these findings were consistent with those observed in the Western blot analysis, except for the SF1-treated cervospheres, in which the Notch1 gene and protein expression exhibited a reciprocal change.

## Discussion

Due to the fundamental implications of CSCs on cancer progression, developing novel approaches to eradicate these cells has become a significant area of cancer research (4, 20). To screen for potential drug sensitivity in CSCs, isolation and enrichment of the cells are crucial (21). In this study, the tumorsphere culture method, which involves cultivating cells under non-adherent and serum-free conditions, has been adopted to induce cell stemness in the SiHa cell line (22). Under these culture conditions, only a small minority of parental cells could survive and develop into spherical clusters (cervospheres), an important characteristic of CSCs (23). Unlike differentiated cancer cells, CSCs can grow independently of a solid surface due to their capacity to avoid anoikis, a type of cell death caused by a loss of cell adhesion by synthesising higher levels of growth factors and extracellular matrix receptors (24).

CSCs from primary tumours or cancer cell lines are primarily characterised by increased sphere- or colony-forming ability *in vitro*, high expression of stem-related biomarkers, and enhanced capacity to form tumours *in vivo* (25). In the present work, serial propagations of the cervospheres revealed that they could regenerate and maintain their sphere formation for an extended period. The high SFE observed in the first generation can be explained by the idea that some cells in the primary or first generation culture, such as progenitor cells, can form short-lived spheres when transferred from adherent to non-adherent cultures (24). Our findings aligned with prior studies indicating that SiHa cervospheres exhibited a relatively constant SFE over five passages, averaging 6% to 8% (26). Furthermore, the ability of the cervospheres to maintain sphere formation throughout multiple generations is closely linked to their capacity for self-renewal, a key CSC characteristic (22).

The stemness properties of the cervospheres were also evaluated by examining the expression profiles of several potential stem cell markers. CD49f and CK17 are surface proteins abundant in the cervical epithelium’s basal layer or reserve cells and identified as the primary receptor proteins for HPV targets (27). They are regarded as putative markers for CCSCs and widely used for CSC isolation in cervical carcinoma (28). Under our study conditions, both cervosphere and monolayer cultures exhibited high percentages of CD49f- and CK17-positive cells, with no significant difference. These findings contradicted the majority of the literature, which has shown that the proportions of CD49f- and CK17-positive cells are considerably higher in CCSC-enriched cultures than in their parental monolayer counterparts (26–28). However, few studies have found that the expression of CD49f and CK17 in cervical tumours can be highly variable. Javed et al. (29) reported high CD49f positivity by flow cytometry in monolayer and sphere cultures (46% to 89% vs 61% to 90%). However, their immunofluorescence findings showed stronger CD49f in monolayer cells, and patient biopsies exhibited highly variable CD49f positivity, indicating that CD49f is context-dependent rather than a definitive CSC marker. While Wang et al. (30) revealed that CSCs and differentiated cells from both primary cervical tumours and the HeLa cell line had comparable levels of CK17 expression, CSCs were found to be more tumorigenic.

Sox2, Nanog, and Oct4 are transcription factors essential for maintaining the CSC self-renewal, pluripotency, and undifferentiation. They are associated with cervical cancer tumorigenesis and progression (31, 32). In the present study, the percentages of Sox2-, Nanog-, and Oct4-positive cells were higher in the cervospheres compared to the parental monolayers. Hence, these findings indicate the existence of a cell population with stem and progenitor cell features in the SiHa-derived cervosphere culture. These findings also align with previous studies that reported higher expression levels and greater proportions of Sox2-, Nanog-, and Oct4-positive cells in CCSC-enriched cultures. Furthermore, the upregulation of these markers has been associated with enhanced cell proliferation, clonogenicity, invasion, chemo/radio resistance, and self-renewal capabilities of the cells (33, 34).

Activation of Notch1 receptor is crucial to cervical cancer development. It has been linked to CSCs, chemoresistance, disease progression, metastases, and poor prognosis (8, 35). The present study demonstrated that cervospheres exhibited higher NICD protein and Notch1 mRNA in untreated cultures than monolayers. These results corroborated those of Low et al. (36), who discovered that tumorspheres derived from HeLa and SiHa cells had elevated Notch1 expression, and its upregulation was associated with resistance to radiation therapy. In a similar vein, other studies found that spheres derived from cervical cancer cell lines and primary tumours exhibited higher levels of Notch1, cleaved Notch1, and Notch target gene Hes1, which led to enhanced stemness markers, increased capacity for self-renewal, and the ability to propagate tumours in nude mice (37, 38). Hence, these findings suggest that Notch1 is important in regulating CCSC stemness.

In the previous study, we demonstrated that SF1 effectively inhibits the viability and stemness properties of the cervosphere *in vitro* and *in vivo* (18). Hence, the current studies further examined whether Notch1 plays a role in SF1’s suppression of the CCSC-like cells. Interestingly, SF1 was found to reduce NICD protein levels in cervospheres, yet did not suppress *Notch1* mRNA expression. Instead, a modest increase in transcript levels was observed. This apparent discrepancy between protein and mRNA abundance is not uncommon. It may arise from post-transcriptional or post-translational regulation, differences in protein stability, or alterations in proteolytic processing of the full-length receptor (39, 40). Since NICD is generated through γ-secretase–mediated cleavage of membrane-bound Notch1, SF1 may inhibit Notch1 signalling by promoting proteasomal degradation of NICD or by interfering with its generation from the precursor receptor, rather than by downregulating *Notch1* transcription. Such a mechanism has been observed with other phytochemicals, for example, withaferin A, which reduced NICD protein abundance while increasing *Notch1* mRNA expression in breast cancer cells (41). It is also possible that the reduction in NICD triggers a compensatory feedback loop that upregulates *Notch1* transcription, a phenomenon described in other contexts of Notch pathway inhibition (42). Hence, further mechanistic studies, including γ-secretase activity assays and proteasome inhibition experiments, are warranted to delineate the precise mode of NICD suppression by SF1.

On the contrary, cisplatin-induced upregulation of Notch1 expression in the cervospheres at both the protein and mRNA levels. These findings align with previous studies, which reported that cisplatin promoted the activation of Notch signalling in cervical cancer cell lines, including HeLa and SiHa (43). Cisplatin can trigger the release of active Notch from the cell membrane via activation of the p53 protein through the DNA damage signal or by increasing γ-secretase protease secretion (44, 45). Furthermore, cisplatin-induced Notch1 activation was found to be responsible for the enrichment of CSCs and multidrug resistance in non-small cell lung cancer (46). Conversely, inhibiting Notch signalling improved cells’ susceptibility to cisplatin and enhanced cisplatin-induced DNA damage and apoptosis in cancer cells (43, 45). Taken together, the results of this study demonstrate that SF1 possessed anticancer properties in CCSC-like cells by inhibiting Notch1 signalling, as opposed to cisplatin. SF1 has been previously characterised using Fourier Transform Infrared Spectroscopy (FTIR) and Liquid Chromatography-Mass Spectrometry (LC-MS), and revealed the presence of alkaloids with functional group amines as the most abundant phytochemical class (16). Hence, alkaloids in SF1 may be responsible for its anti-tumour activities against CCSCs. While there is extensive documentation on the anticancer properties of alkaloids, research on their effects against CSCs is scarce. Therefore, the results of this investigation may shed light on the capacity of plant alkaloids to selectively target CSCs.

## Conclusion

SF1, a standardised, semi-purified fraction derived from *C. nutans* leaves, was found to modulate Notch1 signalling in cervospheres, as indicated by reduced NICD protein levels and altered *Notch1* mRNA expression. Although the exact mechanisms remain elucidated, these effects may contribute to its previously reported anti-CSC properties. Overall, the findings support SF1 as a potential natural agent for targeting pathways associated with cervical cancer stem-like cells.

## References

[1] Singh D, Vignat J, Lorenzoni V, Eslahi M, Ginsburg O, Lauby-Secretan B, Arbyn M, Basu P, Bray F, Vaccarella S (2023). Global estimates of incidence and mortality of cervical cancer in 2020: a baseline analysis of the WHO Global Cervical Cancer Elimination Initiative. Lancet Glob Health.

[2] Kumar V, Bauer C, Stewart JH (2023). TIME Is ticking for cervical cancer. Biology.

[3] Dawood S, Austin L, Cristofanilli M (2014). Cancer stem cells: implications for cancer therapy. Oncology (Williston Park).

[4] Di Fiore R, Suleiman S, Drago-Ferrante R, Subbannayya Y, Pentimalli F, Giordano A (2022). Cancer stem cells and their possible implications in cervical cancer: a short review. Int J Mol Sci.

[5] Chu DT, Nguyen TT, Tien NLB, Tran DK, Jeong JH, Anh PG (2020). Recent progress of stem cell therapy in cancer treatment: molecular mechanisms and potential applications. Cells.

[6] Venkatesh V, Nataraj R, Thangaraj GS, Karthikeyan M, Gnanasekaran A, Kaginelli SB (2018). Targeting Notch signalling pathway of cancer stem cells. Stem Cell Investig.

[7] Li Y, Wicha MS, Schwartz SJ, Sun D (2011). Implications of cancer stem cell theory for cancer chemoprevention by natural dietary compounds. J Nutr Biochem.

[8] Gharaibeh L, Elmadany N, Alwosaibai K, Alshaer W (2020). Notch1 in cancer therapy: possible clinical implications and challenges. Mol Pharmacol.

[9] Rodrigues C, Joy LR, Sachithanandan SP, Krishna S (2019). Notch signalling in cervical cancer. Exp Cell Res.

[10] Bajaj J, Maliekal TT, Vivien E, Pattabiraman C, Srivastava S, Krishnamurthy H (2011). Notch signaling in CD66+ cells drives the progression of human cervical cancers. Cancer Res.

[11] Maliekal TT, Bajaj J, Giri V, Subramanyam D, Krishna S (2008). The role of Notch signaling in human cervical cancer: implications for solid tumors. Oncogene.

[12] Alam A, Ferdosh S, Ghafoor K, Hakim A, Juraimi AS, Sarker ZI (2016). Clinacanthus nutans: a review of the medicinal uses, pharmacology and phytochemistry. Asian Pac J Trop Med.

[13] Ng PY, Chye SM, Ng CH, Koh RY, Tiong YL, Pui LP (2017). Clinacanthus nutans hexane extracts induce apoptosis through a caspase-dependent pathway in human cancer cell lines. Asian Pac J Cancer Prev.

[14] Yong YK, Tan JJ, Teh SS, Mah SH, Ee GCL, Chiong HS (2013). Clinacanthus nutans extracts are antioxidant with antiproliferative effect on cultured human cancer cell lines. Evid Based Complement Alternat Med.

[15] Zainuddin NASN, Hassan NFN, Zakaria Y, Muhammad H, Othman NH (2019). Semi-purified fraction of Clinacanthus nutans induced apoptosis in human cervical cancer, SiHa cells via upregulation of Bax and down-regulation of Bcl-2. Sains Malays.

[16] Zainuddin NASN, Muhammad H, Hassan NFN, Othman NH, Zakaria Y (2020). Clinacanthus nutans standardized fraction arrested SiHa cells at G1/S and induced apoptosis via upregulation of p53. J Pharm Bioallied Sci.

[17] Zainuddin NASN, Muhammad H, Hassan NFN, Zakaria Y (2024). Growth inhibition of standardized amine fraction from Clinacanthus nutans on mice xenograft model for human cervical cancer. Mal J Med Health Sci.

[18] Ismail F, Zakaria Y, Isa MLM, Hassan NFN, Cheng TS (2024). SF1: a standardised fraction of Clinacanthus nutans that inhibits the stemness properties of cancer stem-like cells derived from cervical cancer. Sains Malays.

[19] Chen SF, Chang YC, Nieh S, Liu CL, Yang CY, Lin YS (2012). Nonadhesive culture system as a model of rapid sphere formation with cancer stem cell properties. PLoS One.

[20] Bighetti-Trevisan RL, Sousa LO, Castilho RM, Almeida LO (2019). Cancer stem cells: powerful targets to improve current anticancer therapeutics. Stem Cells Int.

[21] Gilbert CA, Ross AH (2009). Cancer stem cells: cell culture, markers, and targets for new therapies. J Cell Biochem.

[22] Lee CH, Yu CC, Wang BY, Chang WW (2016). Tumorsphere as an effective in vitro platform for screening anticancer stem cell drugs. Oncotarget.

[23] Bielecka ZF, Maliszewska-Olejniczak K, Safir IJ, Szczylik C, Czarnecka AM (2017). Three-dimensional cell culture model utilization in cancer stem cell research. Biol Rev Camb Philos Soc.

[24] Bahmad HF, Cheaito K, Chalhoub RM, Hadadeh O, Monzer A, Ballout F (2018). Sphere-formation assay: three-dimensional in vitro culturing of prostate cancer stem/progenitor sphere-forming cells. Front Oncol.

[25] Tirino V, Desiderio V, Paino F, Papaccio G, De Rosa M (2012). Methods for cancer stem cell detection and isolation. Methods Mol Biol.

[26] López J, Poitevin A, Mendoza-Martínez V, Pérez-Plasencia C, García-Carrancá A (2012). Cancer-initiating cells derived from established cervical cell lines exhibit stem-cell markers and increased radioresistance. BMC Cancer.

[27] Bigoni-Ordóñez GD, Ortiz-Sánchez E, Rosendo-Chalma P, Valencia-González HA, Aceves C, García-Carrancá A (2018). Molecular iodine inhibits the expression of stemness markers on cancer stem-like cells of established cell lines derived from cervical cancer. BMC Cancer.

[28] Ortiz-Sánchez E, Santiago-López L, Cruz-Domínguez VB, Toledo-Guzmán ME, Hernández-Cueto D, Muñiz-Hernández S (2016). Characterization of cervical cancer stem cell-like cells: phenotyping, stemness, and human papilloma virus co-receptor expression. Oncotarget.

[29] Javed S, Sharma BK, Sood S, Sharma S, Bagga R, Bhattacharyya S (2018). Significance of CD133 positive cells in four novel HPV-16 positive cervical cancer-derived cell lines and biopsies of invasive cervical cancer. BMC Cancer.

[30] Wang Y, Wang M, Zeng Q, Lv Y, Bao B (2017). Isolation and biological characteristics of human cervical cancer side population cells. Int J Clin Exp Pathol.

[31] Shen L, Huang X, Xie X, Su J, Yuan J, Chen X (2014). High expression of SOX2 and OCT4 indicates radiation resistance and an independent negative prognosis in cervical squamous cell carcinoma. J Histochem Cytochem.

[32] Ye F, Zhou C, Cheng Q, Shen J, Chen H (2008). Stem-cell-abundant proteins Nanog, Nucleostemin and Musashi1 are highly expressed in malignant cervical epithelial cells. BMC Cancer.

[33] Zhou X, Yue Y, Wang R, Gong B, Duan Z (2017). MicroRNA-145 inhibits tumorigenesis and invasion of cervical cancer stem cells. Int J Oncol.

[34] Wang L, Guo H, Lin C, Yang L, Wang X (2014). Enrichment and characterization of cancer stem-like cells from a cervical cancer cell line. Mol Med Rep.

[35] Sun Y, Zhang R, Zhou S, Ji Y (2015). Overexpression of Notch1 is associated with the progression of cervical cancer. Oncol Lett.

[36] Low HY, Lee YC, Lee YJ, Wang HL, Chen YI, Chien PJ (2020). Reciprocal regulation between indoleamine 2,3-dioxigenase 1 and Notch1 involved in radiation response of cervical cancer stem cells. Cancers (Basel).

[37] Tyagi A, Vishnoi K, Mahata S, Verma G, Srivastava Y, Masaldan S (2016). Cervical cancer stem cells selectively overexpress HPV oncoprotein E6 that controls stemness and self-renewal through upregulation of HES1. Clin Cancer Res.

[38] Prabakaran DS, Muthusami S, Sivaraman T, Yu JR, Park WY (2019). Silencing of FTS increases radiosensitivity by blocking radiation-induced Notch1 activation and spheroid formation in cervical cancer cells. Int J Biol Macromol.

[39] Nie L, Wu G, Zhang W (2006). Correlation of mRNA expression and protein abundance affected by multiple sequence features related to translational efficiency in Desulfovibrio vulgaris: a quantitative analysis. Genetics.

[40] Schwanhäusser B, Busse D, Li N, Dittmar G, Schuchhardt J, Wolf J (2011). Global quantification of mammalian gene expression control. Nature.

[41] Lee J, Sehrawat A, Singh SV (2012). Withaferin A causes activation of Notch2 and Notch4 in human breast cancer cells. Breast Cancer Res Treat.

[42] Andersson ER, Sandberg R, Lendahl U (2011). Notch signaling: simplicity in design, versatility in function. Development.

[43] Li S, Ren B, Shi Y, Gao H, Wang J, Xin Y (2019). Notch1 inhibition enhances DNA damage induced by cisplatin in cervical cancer. Exp Cell Res.

[44] Meng RD, Shelton CC, Li YM, Qin LX, Notterman D, Paty P (2009). Gamma-secretase inhibitors abrogate oxaliplatin-induced activation of the Notch-1 signaling pathway in colon cancer cells resulting in enhanced chemosensitivity. Cancer Res.

[45] Qu J, Wang Y, Yang Y, Liu J (2017). Targeting Notch-1 reverses cisplatin chemosensitivity in ovarian cancer cells by upregulation of PUMA. Int J Clin Exp Med.

[46] Wang L, Liu X, Ren Y, Zhang J, Chen J, Zhou W (2017). Cisplatin-enriching cancer stem cells confer multidrug resistance in non-small cell lung cancer via enhancing TRIB1/HDAC activity. Cell Death Dis.

